# Skeletal Muscle Volume Is an Independent Predictor of Survival after Sorafenib Treatment Failure for Hepatocellular Carcinoma

**DOI:** 10.3390/cancers13092247

**Published:** 2021-05-07

**Authors:** Issei Saeki, Takahiro Yamasaki, Yurika Yamauchi, Taro Takami, Tomokazu Kawaoka, Shinsuke Uchikawa, Akira Hiramatsu, Hiroshi Aikata, Reo Kawano, Kazufumi Kobayashi, Takayuki Kondo, Sadahisa Ogasawara, Tetsuhiro Chiba, Kazuaki Chayama, Naoya Kato, Isao Sakaida

**Affiliations:** 1 Department of Gastroenterology and Hepatology, Yamaguchi University Graduate School of Medicine, Yamaguchi 755-8505, Japan; issaeki@yamaguchi-u.ac.jp (I.S.); g030ub@yamaguchi-u.ac.jp (Y.Y.); t-takami@yamaguchi-u.ac.jp (T.T.); sakaida@yamaguchi-u.ac.jp (I.S.); 2 Department of Oncology and Laboratory, Yamaguchi University Graduate School of Medicine, Yamaguchi 755-8505, Japan; 3 Department of Gastroenterology and Metabolism, Graduate School of Biomedical and Health Sciences, Hiroshima University, Hiroshima 734-8551, Japan; kawaokatomo@hiroshima-u.ac.jp (T.K.); shinuchi@hiroshima-u.ac.jp (S.U.); akirah@hiroshima-u.ac.jp (A.H.); aikata@hiroshima-u.ac.jp (H.A.); chayama@hiroshima-u.ac.jp (K.C.); 4 Clinical Research Center in Hiroshima, Hiroshima University Hospital, Hiroshima 734-8551, Japan; rkawano@hiroshima-u.ac.jp; 5 Department of Gastroenterology, Graduate School of Medicine, Chiba University, Chiba 260-8667, Japan; kobayashi-kazufumi@chiba-u.jp (K.K.); takakon@chiba-u.jp (T.K.); ogasawaras@chiba-u.jp (S.O.); chibat@chiba-u.jp (T.C.); kato.naoya@chiba-u.jp (N.K.); 6 Translational Research and Development Center, Chiba University Hospital, Chiba 260-8667, Japan

**Keywords:** hepatocellular carcinoma, muscle depletion, post-progression survival, sorafenib

## Abstract

**Simple Summary:**

Skeletal muscle volume has been reported as a prognostic factor for patients with hepatocellular carcinoma receiving sorafenib. In this study, we show that skeletal muscle volume is not only a predictor of overall survival but also of post-progression survival, which represents survival time following confirmation of progressive disease. We may be able to prolong survival by upregulating skeletal muscle volume, especially in hepatocellular carcinoma patients with skeletal muscle depletion.

**Abstract:**

Few studies exist on the relationship between post-progression survival (PPS) and skeletal muscle volume in hepatocellular carcinoma (HCC) patients receiving sorafenib. This study aimed to analyze the effects of muscle volume on clinical outcomes. We retrospectively enrolled 356 HCC patients. Various clinical parameters, including skeletal muscle index, were analyzed as predictors of overall survival (OS), progression-free survival (PFS), and PPS. Patients with high muscle volume showed longer survival or PPS than those with low muscle volume (median survival time: 12.8 vs. 9.5 months, *p* = 0.005; median PPS: 8.2 vs. 6.3 months, *p* = 0.015); however, no differences in PFS were found. Multivariate analysis indicated that muscle volume was an independent predictor of PPS and OS. Skeletal muscle volume was a PPS predictor in HCC patients receiving sorafenib. Therefore, survival can be prolonged by the upregulation of skeletal muscle volume, especially in HCC patients with skeletal muscle depletion.

## 1. Introduction

Numerous molecular-targeted agents (MTAs) and immune checkpoint inhibitors (ICIs) have been introduced for treating hepatocellular carcinoma (HCC). Sorafenib was approved in 2007 as a first-line drug for the systemic treatment of advanced HCC [1] and has played a significant role in the development of clinical trials. However, compared to treatment with sorafenib, treatment with a combination of atezolizumab and bevacizumab led to a greater improvement in overall survival (OS) and progression-free survival (PFS) [2]; thus, this combination therapy can be considered as the first-line therapy for advanced HCC. Although OS is commonly used as the primary outcome in clinical trials for HCC [1,2,3,4,5,6], post-progression survival (PPS) is an important factor for prolonging OS. Moreover, the RESORCE study and a sub-analysis of the REFLECT study demonstrated that sequential therapy improved survival in patients who were refractory to the first-line therapy [3,7]. Furthermore, PPS was highly correlated with OS in HCC patients receiving sorafenib [8]. Several predictive biomarkers of OS are reported in HCC patients receiving sorafenib, but there are no useful PPS predictors in such patients [9]. It is possible to evaluate these in HCC patients receiving sorafenib who have long follow-up periods.

Sarcopenia was first defined in 1989 as age-related skeletal muscle depletion [10,11]; studies have shown that it is caused by various cancers, including HCC [12,13,14]. Sarcopenia diagnosis criteria include impaired physical performance and loss of muscle volume and strength in the aged population [15,16]. However, skeletal muscle depletion has been the most commonly used definition for sarcopenia [13,14] and has been reported as a poor prognostic factor in HCC patients receiving sorafenib [17,18,19]. To the best of our knowledge, there are no studies on the relationship between PPS and skeletal muscle volume before sorafenib treatment for HCC.

In this multicenter study, we retrospectively investigated the effects of skeletal muscle volume on clinical outcomes such as OS, PFS, and PPS in HCC patients receiving sorafenib.

## 2. Materials and Methods

### 2.1. Study Population

Patients who received treatment with sorafenib at three hospitals in Japan (the Chiba University Hospital, Hiroshima University Hospital, and Yamaguchi University Hospital) between April 2009 and July 2016 were included in this study. HCC diagnosis was based on radiological or pathological findings and expression of tumor markers (alpha-fetoprotein and des-gamma-carboxy prothrombin). The inclusion criteria were as follows: (i) unresectable HCC confirmed by a pathological or imaging diagnosis; (ii) stage B or C tumor according to the Barcelona Clinic Liver Cancer (BCLC) staging system [20]; and (iii) computed tomography (CT) data available for up to the third lumbar vertebra before sorafenib treatment. Patients with incomplete data were excluded. As shown in Figure S1, a total of 546 patients received sorafenib during the study period. Among these, clinical data and information on skeletal muscle volume were lacking in 152 patients, while 38 did not undergo radiological imaging during treatment. Thus, OS or PFS were evaluated only in 356 patients. Of these, 36 patients did not develop radiological tumor progression until the last follow-up date; hence, PPS was analyzed in 320 patients. This study was approved by the Institutional Review Board of the Yamaguchi University Hospital (H30-042) and those of the two other institutions and was performed in accordance with the ethical principles of the 1975 Declaration of Helsinki. Informed consent was not obtained due to the retrospective study design.

### 2.2. Treatment

In principle, the daily starting dose of sorafenib was 800 mg, but it was reduced depending on the patient’s liver function and/or general status. Sorafenib treatment was continued until the patient experienced tumor progression, unacceptable adverse events, or death. Post-progression treatments, such as transcatheter arterial chemoembolization (TACE), hepatic resection, hepatic arterial infusion chemotherapy (HAIC), and other systemic therapies were performed as per the patient’s liver function, tumor burden, and performance status, as well as at the discretion of each hospital.

### 2.3. Assessment of Skeletal Muscle Volume

All patients underwent CT within 1 month before sorafenib treatment. Radiological data were collected as Digital Imaging and Communication in Medicine data at the Yamaguchi University Hospital. At this hospital, skeletal muscle volume was measured at the level of the third lumbar vertebra using an AZE 3D workstation (AZE Virtual Place Raijin; AZE Ltd., Tokyo, Japan) and was categorized on the basis of a CT radiodensity value of −29 to +150 HU [21]. Muscle area was standardized as the square of height, and skeletal muscle index (SMI) was calculated by dividing the skeletal muscle mass by the square of height. Skeletal muscle volume was classified into high (H-MV) and low (L-MV) muscle volume using the median SMI in males or females as the threshold.

### 2.4. Evaluation of Treatment Response

Treatment response was evaluated according to the Response Evaluation Criteria in Solid Tumors [22] and based on dynamic CT or dynamic magnetic resonance imaging every 2–3 months. The best response was used for response evaluation.

### 2.5. Statistical Analyses

Continuous variables are expressed as medians and interquartile ranges (IQRs). Between-group comparisons were performed using the chi-squared test or the Fisher’s exact test. OS, PFS, and PPS were calculated using the Kaplan–Meier method. OS was defined as the interval from the start of sorafenib treatment to death, last visit, or last follow-up. PFS was defined as the interval from the start of sorafenib treatment to the first radiological confirmation of progressive disease (PD) or death. PPS was defined as the interval from PD confirmation to death, last visit, or last follow-up. The follow-up period ended on 31 March 2018. A total of 273 patients died during the study period, and Spearman’s test was used to estimate the correlation between OS and PFS/PPS in them.

To define the OS, PFS, and PPS predictive factors, we assessed the following baseline parameters before sorafenib treatment: age (<70 years or ≥70 years), sex (male/female), body mass index (BMI) (≥22 kg/m^2^ or <22 kg/m^2^), Eastern Cooperative Oncology Group performance status (ECOG-PS) (0, 1, or 2), Child–Pugh class (A/B), tumor number (<8 or ≥8), tumor size (<35 mm or ≥35 mm), macrovascular invasion (MVI) (absence/presence), extrahepatic spread (EHS) (absence/presence), and skeletal muscle volume (high/low). Median values were used as the threshold for age, tumor size, and tumor number. The threshold for BMI, which is considered standard due to having the lowest morbidity [23], was used. Furthermore, to analyze the prognostic parameters of OS in the 320 patients who were refractory to sorafenib treatment, we included two factors in addition to the aforementioned parameters: disease control with sorafenib (yes/no) and post-sorafenib therapy (yes/no). Univariate and multivariate analyses of the predictive factors were performed using the Cox proportional hazard model and logistic regression analysis; the results are presented as hazard ratios (HRs) or odds ratios (ORs) with 95% confidence intervals (CIs). All statistical analyses were performed using JMP Pro 15 software (SAS Institute Inc., Cary, NC, USA). Statistical significance was defined at a *p*-value < 0.05.

## 3. Results

### 3.1. Patient Characteristics

The patients’ profiles are summarized in Table 1. A total of 310 and 46 patients had a Child–Pugh class of A and B, respectively. In terms of BCLC staging, 78 and 278 patients were classified as having stage B and C tumors, respectively. The median SMI was 45.3 cm^2^/m^2^ in males and 38.3 cm^2^/m^2^ in females. Therefore, L-MV was defined as an SMI of <45 cm^2^/m^2^ in males and <38 cm^2^/m^2^ in females.

### 3.2. Treatment Response and Its Predictors

A total of 16 (4.5%), 197 (55.3%), and 143 (40.2%) patients showed partial response (PR), stable disease (SD), and PD, respectively. The objective response rate (ORR) and disease control rate (DCR) were 4.5% and 59.8%, respectively. Univariate and multivariate analyses demonstrated that absence of MVI contributed to disease control after treatment with sorafenib (OR: 1.750, 95% CI: 1.028–2.978, *p* = 0.039), but skeletal muscle volume was not a significant predictor of disease control after treatment with sorafenib (Table S1).

### 3.3. OS, PPS, and PFS According to Skeletal Muscle Volume

**Figure 1:**
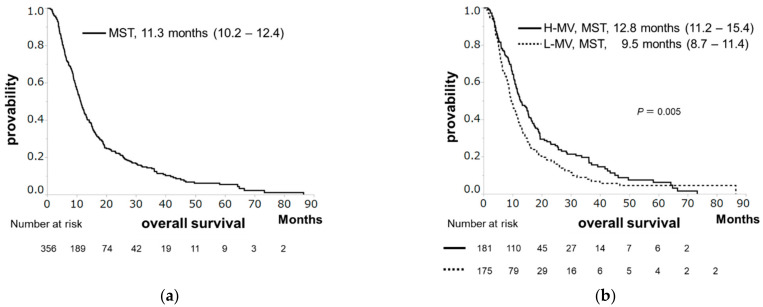
Overall survival (OS) in hepatocellular carcinoma patients receiving sorafenib. (**a**) The OS rates at 1, 2, and 3 years are 46.5%, 22.2%, and 13.9 %, respectively, with a median survival time (MST) of 11.3 months. (**b**) OS according to skeletal muscle volume. Patients with high muscle volume (H-MV) showed significantly longer survival than those with low muscle volume (L-MV) (MST: 12.8 vs. 9.5 months, *p* = 0.005).

**Figure 2:**
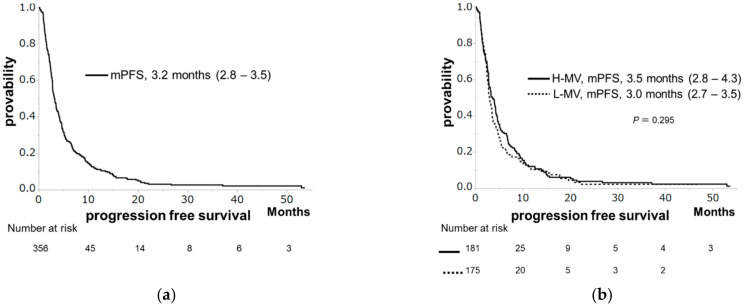
Progression-free survival (PPS) in hepatocellular carcinoma patients receiving sorafenib. (**a**) The PFS rates at 1 and 2 years are 11.2% and 3.1 %, respectively, with a median PFS of 3.2 months. (**b**) PFS according to skeletal muscle volume. No significant difference in PFS can be observed between patients with high muscle volume (H-MV) and those with low muscle volume (L-MV) (median PFS: 3.5 vs. 3.0 months, *p* = 0.295).

**Figure 3:**
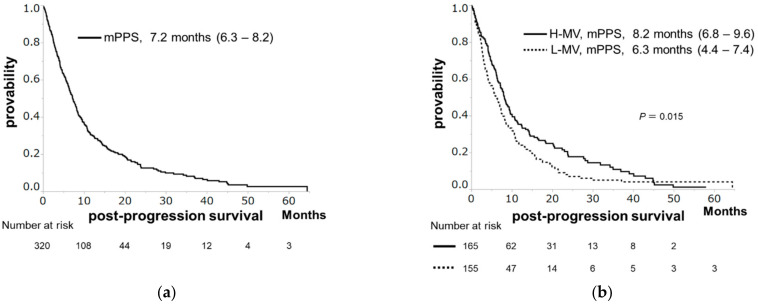
Post-progression survival (PPS) in hepatocellular carcinoma patients receiving sorafenib. (**a**) The PPS rates at 1 and 2 years are 30.3% and 12.6 %, respectively, with a median PPS of 7.2 months. (**b**) PPS according to skeletal muscle volume. Patients with high muscle volume (H-MV) showed significantly longer PPS than those with low muscle volume (L-MV) (median PPS, 8.2 vs. 6.3 months, *p* = 0.015).

The median survival time (MST), median PFS, and median PPS were 11.3, 3.2, and 7.2 months, respectively (Figure 1a, Figure 2a and Figure 3a). Patients with H-MV showed significantly longer survival or PPS than those with L-MV (MST: 12.8 vs. 9.5 months, *p* = 0.005, Figure 1b; median PPS: 8.2 vs. 6.3 months, *p* = 0.015, Figure 3b). However, there was no significant difference in PFS between patients with H-MV and those with L-MV (median PFS: 3.5 vs. 3.0 months, *p* = 0.295, Figure 2b). We compared the H-MV and L-MV characteristics between patients followed for OS/PFS (*n* = 356) and those followed for PPS (*n* = 320) analysis. There were no significant differences in the factors considered, except for BMI, tumor number, and MVI (Tables S2 and S3).

### 3.4. Correlation between OS and PFS/PPS

We analyzed the correlation between OS and PFS/PPS in 273 patients who died during the study period. PPS was strongly correlated with OS (R = 0.908, *p* < 0.001), while PFS was moderately correlated with OS (R = 0.642, *p* < 0.001) (Figure S2).

### 3.5. Predictors of PFS

On multivariate analysis, tumor number < 8 (HR: 0.611, 95% CI: 0.482–0.770, *p* < 0.001) and the absence of EHS (HR: 0.733, 95% CI: 0.582–0.924, *p* = 0.008) were significant predictors of PFS, whereas skeletal muscle volume was not a significant predictor of PFS (*p* = 0.255) (Table S4).

### 3.6. Predictors of PPS or OS

On multivariate analysis, six factors—male sex (HR: 0.672, 95% CI: 0.494–0.914, *p* = 0.011), Child–Pugh class A (HR: 0.612, 95% CI: 0.421–0.890, *p* = 0.010), tumor number < 8 (HR: 0.656, 95% CI: 0.509–0.844, *p* = 0.001), absence of MVI (HR: 0.720, 95% CI: 0.528–0.982, *p* = 0.038), absence of EHS (HR: 0.674, 95% CI: 0.521–0.872, *p* = 0.003), and H-MV (HR: 0.698, 95% CI: 0.509–0.959, *p* = 0.027)—were found to be significant predictors of PPS (Table 2).

Furthermore, as shown in Table 3, multivariate analysis indicated that these factors were also significant predictors of OS: male sex (HR: 0.661, 95% CI: 0.491–0.889, *p* = 0.006), Child–Pugh class A (HR: 0.701, 95% CI: 0.496–0.992, *p* = 0.045), tumor number <8 (HR: 0.591, 95% CI: 0.465–0.751, *p* < 0.001), absence of MVI (HR: 0.678, 95% CI: 0.506–0.907, *p* = 0.009), absence of EHS (HR: 0.600, 95% CI: 0.470–0.766, *p* < 0.001), and H-MV (HR: 0.666, 95% CI: 0.501–0.886, *p* = 0.005).

### 3.7. Prognostic Factor of OS

On multivariate analysis, the following six factors were found to be independent prognostic factors of OS: sex (HR: 0.717, 95% CI: 0.524–0.980, *p* = 0.037), tumor number (HR: 0.615, 95% CI: 0.476–0.793, *p* < 0.001), EHS (HR: 0.684, 95% CI: 0.527–0.887, *p* = 0.004), skeletal muscle volume (HR: 0.545, 95% CI: 0.393–0.755, *p* < 0.001), disease control (HR: 0.398, 95% CI: 0.307–0.516, *p* < 0.001), and post-sorafenib therapy (HR: 0.610, 95% CI: 0.472–0.789, *p* < 0.001) (Table 4). Additionally, the results of the post-sorafenib therapies are shown in Figure S3.

## 4. Discussion

Several studies on lung, gastric, colorectal, breast, and ovarian cancers have demonstrated that OS, which is considered as the primary outcome in clinical trials on cancer patients who are ineligible for therapy, is strongly correlated with PPS [24,25,26,27,28]. Even in patients with HCC, the same recent studies showed that PPS was strongly correlated with OS. However, PFS was not as strongly correlated with OS as PPS was in HCC patients receiving sorafenib [8] or systemic therapy in randomized controlled trials [29]. This multicenter study also demonstrated a strong correlation between OS and PPS (R = 0.908) in a large population of HCC patients (*n* = 273) receiving sorafenib, and this correlation was stronger than that between OS and PFS (R = 0.642). These results suggest that post-progression treatment after sorafenib failure can also be an important factor for prolonging OS. Currently, there are several MTAs for HCC patients who are resistant to sorafenib, and these drugs reportedly increase survival in these patients [3,5,6]. In this study, TACE and HAIC were used as salvage options for post-sorafenib therapy. It has been previously reported that subsequent therapy, including TACE and HAIC, contributes to prolonging PPS after sorafenib therapy [30,31,32]. We also demonstrate that post-sorafenib therapy is an independent prognostic factor for OS, similar to the PPS prognostic factor (Table S5 and Figure S4). Therefore, these results indicate that sequential therapy after sorafenib failure is important for prolonging PPS.

Previous reports demonstrated that skeletal muscle depletion is a prognostic factor for poor OS in HCC patients receiving sorafenib [17,18,19]. As skeletal muscle volume is not associated with time to progression [17], which is associated with PFS, it has been suggested that it is associated with PPS. However, there have been few studies on the relationship between skeletal muscle volume and PPS [33]. Although a recent report showed that PPS is significantly associated with pre-sarcopenia at the time of PD in HCC patients receiving sorafenib, the relationship between skeletal muscle volume before sorafenib treatment and PPS has not been established [33]. This is the first study to demonstrate that skeletal muscle volume before sorafenib treatment is a significant predictor of PPS, but not PFS. Additionally, as both skeletal muscle volume and post-sorafenib therapy were found to be independent prognostic factors of PPS and OS, a difference in L-MV and H-MV may also be an important prognostic factor regardless of the presence of post-sorafenib therapy.

The annual rates of skeletal muscle volume decline in cirrhotic patients were reported to be 1.3%, 3.5%, and 6.1% for Child–Pugh classes A, B, and C, respectively [34]. In contrast, our previous study found that skeletal muscle volume decreased by 5.8% 3 months after starting sorafenib treatment, regardless of muscle volume [19]. Another study demonstrated a significant loss of skeletal muscle volume regardless of the presence of disease progression in HCC patients treated with sorafenib or lenvatinib [35]. Thus, compared to treatment with HAIC, treatment with sorafenib may markedly reduce skeletal muscle volume [36] and significantly worsen liver function [37]. Moreover, although skeletal muscle volume is not associated with treatment response to sorafenib and PFS, patients may still require an adequate amount of skeletal muscle volume to receive sorafenib and maintain a Child–Pugh class of A after sorafenib therapy. Therefore, skeletal muscle volume may be considered as an energy reservoir for prolonging PPS in HCC patients receiving sorafenib.

In the era of MTAs, compared to first-line MTA therapy, sequential therapy using MTAs may decrease skeletal muscle volume more significantly; thus, patients with H-MV may have a survival advantage even without achieving disease control with first-line therapy, because skeletal muscle volume and disease control were found to be independent predictors of OS. In addition, it is possible to improve skeletal muscle volume before and during MTA therapy by exercise and interventional nutritional therapy, including branched-chain amino acid and L-carnitine supplementation [38,39,40,41,42]. However, there have been no studies regarding the efficacy of exercise in HCC patients treated with MTAs. Although further investigation is necessary, we highly expect a combination of exercise and nutritional therapy in HCC patients with L-MV to have a synergetic effect.

There are some limitations to this study. First, this is a multicenter, retrospective cohort study. However, it may be impossible to use the same study design in a multicenter prospective study during the same period using multiple MTAs and/or immune checkpoint inhibitors. We consider that this study minimizes the influence of previous systemic therapies and evaluates clinical course following sorafenib therapy. Second, the median value of SMI in males and females was used as the cutoff value in this study (SMI < 45 cm^2^/m^2^ in males and <38 cm^2^/m^2^ in females). The cutoff values of SMI reportedly vary from 36.2 to 52.4 cm^2^/m^2^ in males and from 29.0 to 39.5 cm^2^/m^2^ in females [43]. The Japan Society of Hepatology (JSH) proposed the criteria for sarcopenia in patients with chronic liver disease [44], wherein skeletal muscle depletion for SMI was defined as a value <42 cm^2^/m^2^ in males and <38 cm^2^/m^2^ in females. However, as the optimal cutoff values for CT imaging, which are equivalent to those for bioelectrical impedance analysis (7.0 kg/m^2^ in males and 5.7 kg/m^2^ in females) based on the Asian Working Group for Sarcopenia criteria [16], were calculated using a receiver-operating characteristic curve analysis of 149 patients with liver cirrhosis or HCC, these cutoff values are provisional. Currently, the cutoff value for SMI in females is the same as the value provided by JSH (38 cm^2^/m^2^), while the cutoff for males is different between this study (45 cm^2^/m^2^) and that given by JSH (42 cm^2^/m^2^). Therefore, our findings suggest that SMI values ranging from 42 to 45 cm^2^/m^2^ in males could constitute a critical range affecting clinical outcomes. Such a range may be important in determining the best cutoff values for skeletal muscle depletion in Japanese patients with HCC. Finally, in this study, post-progression treatment after sorafenib failure mainly used TACE or HAIC, rather than an MTA. The recent trend of post-progression treatment is different from this study, because of the approval of numerous MTAs and ICIs.. Patients with progressive loss of skeletal muscle volume after TACE had a significantly poor prognosis [45,46], whereas skeletal muscle depletion was not associated with OS in patients receiving HAIC [36]. Additionally, as opposed to sorafenib, HAIC was associated with a slight decrease in skeletal muscle volume [36]. Conversely, skeletal muscle volume significantly decreased after the indication of sorafenib or lenvatinib [19,35], although there have been no reports on the relationship between skeletal muscle volume and clinical outcomes in patients receiving a combination of atezolizumab and bevacizumab or MTAs other than those mentioned above. Therefore, we believe that an adequate amount of skeletal muscle volume has a positive impact on the PPS regardless of all treatments.

## 5. Conclusions

This study demonstrates that skeletal muscle volume is a significant predictor of PPS in HCC patients receiving sorafenib. Therefore, upregulation of skeletal muscle volume may improve survival, especially in HCC patients with skeletal muscle depletion.

## Figures and Tables

**Table 1:** Patient characteristics.

Factors		Total (*N* = 356)
Age		69.5 (63.0–75.0)
Sex (male/female)		287/69
Etiology (HCV/HBV/HBV+HCV/NBNC)		175/80/2/99
Body mass index [kg/m^2^]		22.9 (20.8–24.9)
ECOG-PS (0/1/2/3)		314/37/3/2
Child–Pugh class (A/B)		310/46
Barcelona Clinic Liver Cancer stage (B/C)		78/278
Tumor number		8 (2–8)
Tumor size [mm]		35.0 (18.3–65.0)
Macrovascular invasion (−/+)		258/98
Extrahepatic spread (−/+)		167/189
Response (CR/PR/SD/PD)		0/16/197/143
Skeletal mass index	male	45.3 (41.2–50.4)
	female	38.3 (34.0–42.9)
Muscle volume (high/low)		181/175

HCV, Hepatitis C virus; HBV, Hepatitis B virus; NBNC, NonBnonC; ECOG-PS, Eastern Cooperative Oncology Group performance status; CR, domplete response; PR, partial response; SD, stable disease; PD, progressive disease.

**Table 2:** Univariate and multivariate analyses for predictors of post-progression survival (*n* = 320).

Factors	Univariate Analysis			Multivariate Analysis		
	HR	95%CI	*p* Value	HR	95%CI	*p* Value
Age (<70/≥70)	1.135	0.893–1.442	0.300	1.082	0.844–1.386	0.534
Sex (male/female)	0.742	0.549–1.003	0.052	0.672	0.494–0.914	**0.011**
Body mass index [kg/m^2^] (≥22/<22)	0.918	0.720–1.170	0.488	1.155	0.849–1.570	0.360
ECOG-PS (−1/2-)	0.607	0.250–1.476	0.271	0.633	0.257–1.555	0.318
Child–Pugh class (A/B)	0.617	0.432–0.880	**0.008**	0.612	0.421–0.890	**0.010**
Tumor number (<8/≥8)	0.674	0.529–0.859	**0.001**	0.656	0.509–0.844	**0.001**
Tumor size [mm] (<35/≥35)	0.820	0.646–1.041	0.102	0.863	0.661–1.125	0.276
Macrovascular invasion (−/+)	0.614	0.469–0.804	**<0.001**	0.720	0.528–0.982	**0.038**
Extrahepatic spread (−/+)	0.842	0.661–1.070	0.160	0.674	0.521–0.872	**0.003**
Muscle volume (high/low)	0.745	0.586–0.946	**0.016**	0.698	0.509–0.959	**0.027**

ECOG-PS, Eastern Cooperative Oncology Group performance status; HR, hazard ratio; CI, confidence interval. Bold means a *p*-value < 0.05.

**Table 3:** Univariate and multivariate analyses for predictors of overall survival (*n* = 356).

**Factors**	**Univariate Analysis**			**Multivariate Analysis**		
	**HR**	**95%CI**	***p* Value**	**HR**	**95%CI**	***p* Value**
Age (<70/≥70)	1.055	0.840–1.325	0.643	1.002	0.792–1.266	0.989
Sex (male/female)	0.766	0.576–1.019	0.067	0.661	0.491–0.889	**0.006**
Body mass index [kg/m^2^] (≥22/<22)	0.918	0.728–1.158	0.472	1.217	0.919–1.612	0.170
ECOG-PS (−1/2-)	0.578	0.238–1.404	0.266	0.590	0.241–1.445	0.248
Child–Pugh class (A/B)	0.685	0.490–0.956	**0.026**	0.701	0.496–0.992	**0.045**
Tumor number (<8/≥8)	0.625	0.496–0.788	**<0.001**	0.591	0.465–0.751	**<0.001**
Tumor size [mm] (<35/≥35)	0.805	0.640–1.012	0.063	0.863	0.670–1.113	0.256
Macrovascular invasion (−/+)	0.584	0.453–0.754	**<0.001**	0.678	0.506–0.907	**0.009**
Extrahepatic spread (−/+)	0.771	0.612–0.970	**0.027**	0.600	0.470–0.766	**<0.001**
Muscle volume (high/low)	0.721	0.574–0.907	**0.005**	0.666	0.501–0.886	**0.005**

ECOG-PS, Eastern Cooperative Oncology Group performance status; HR, hazard ratio; CI, confidence interval. Bold means a *p*-value < 0.05.

**Table 4:** Univariate and multivariate analyses for prognostic factors of overall survival in patients with progression (*n* = 320).

**Factors**	**Univariate Analysis**			**Multivariate Analysis**		
	**HR**	**95%CI**	***p* Value**	**HR**	**95%CI**	***p* Value**
Age (<70/≥70)	1.092	0.860–1.385	0.471	0.996	0.774–1.282	0.975
Sex (male/female)	0.718	0.531–0.972	0.032	0.717	0.524–0.980	**0.037**
Body mass index [kg/m^2^] (≥22/<22)	0.913	0.716–1.164	0.463	1.193	0.871–1.635	0.271
ECOG-PS (-1/2-)	0.572	0.235–1.3908	0.217	0.570	0.229–1.421	0.228
Child-Pugh class (A /B)	0.691	0.484–0.986	0.041	0.685	0.469–1.002	0.051
Tumor number (<8/≥8)	0.673	0.528–0.858	0.001	0.615	0.476–0.793	**<0.001**
Tumor size [mm] (<35/≥35)	0.774	0.609–0.984	0.037	0.807	0.612–1.063	0.127
Macrovascular invasion (−/+)	0.585	0.446–0.766	<0.001	0.858	0.621–1.185	0.352
Extrahepatic spread (−/+)	0.799	0.627–1.017	0.068	0.684	0.527–0.887	**0.004**
Muscle volume (high/low)	0.704	0.555–0.894	0.004	0.545	0.393–0.755	**<0.001**
Disease control (yes/no)	0.431	0.336–0.552	<0.001	0.398	0.307–0.516	**<0.001**
Post-sorafenib therapy (yes/no)	0.575	0.449–0.736	<0.001	0.610	0.472–0.789	**<0.001**

ECOG-PS, Eastern Cooperative Oncology Group performance status; HR, hazard ratio; CI, confidence interval. Bold means a *p*-value < 0.05.

## Data Availability

The dataset is available from the corresponding author on reasonable request.

## Supplementary Materials

The following are available online at https://www.mdpi.com/article/10.3390/cancers13092247/s1. Figure S1: Enrollment of patients and outcomes, Figure S2: Correlation between overall survival (OS) and either progression-free survival (PFS) or post-progression survival (PPS), Figure S3: Post-sorafenib therapy according to skeletal muscle volume, Figure S4: Post-progression survival according to absence or presence of post-sorafenib therapy, Table S1: Univariate and multivariate analyses of predictors affecting disease control with sorafenib, Table S2: Characteristics of 356 patients with hepatocellular carcinoma according to the skeletal muscle volume, Table S3: Characteristics of 320 patients with hepatocellular carcinoma according to the skeletal muscle volume, Table S4: Univariate and multivariate analyses for predictors of progression-free survival, Table S5: Univariate and multivariate analyses for prognostic factors of post-progression survival.
