# Supplementary figures and images for: Skeletal Muscle Volume Is an Independent Predictor of Survival after Sorafenib Treatment Failure for Hepatocellular Carcinoma
Source: Cancers (Basel). 2021 May 7;13(9):2247. doi: 10.3390/cancers13092247 (PMC8124673; doi:10.3390/cancers13092247)

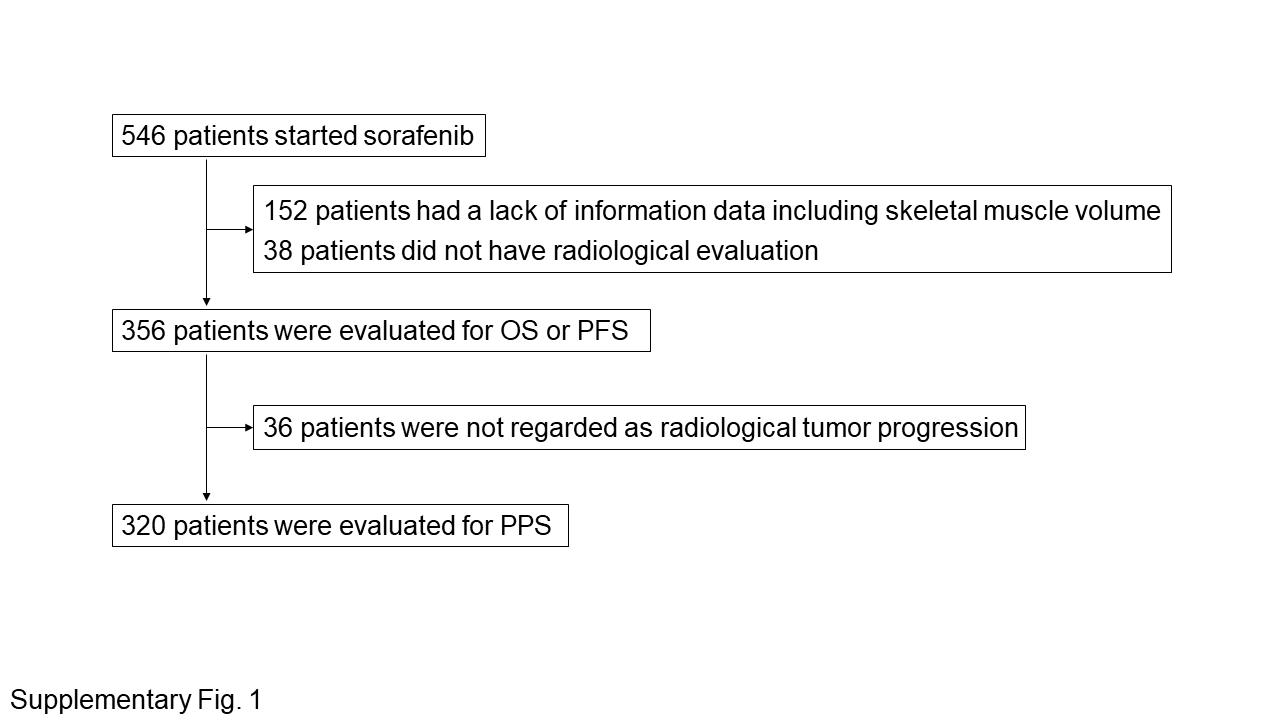

Supplement: Supplementary file 1 [file cancers-13-02247-s001.zip › Fig S1.TIF]

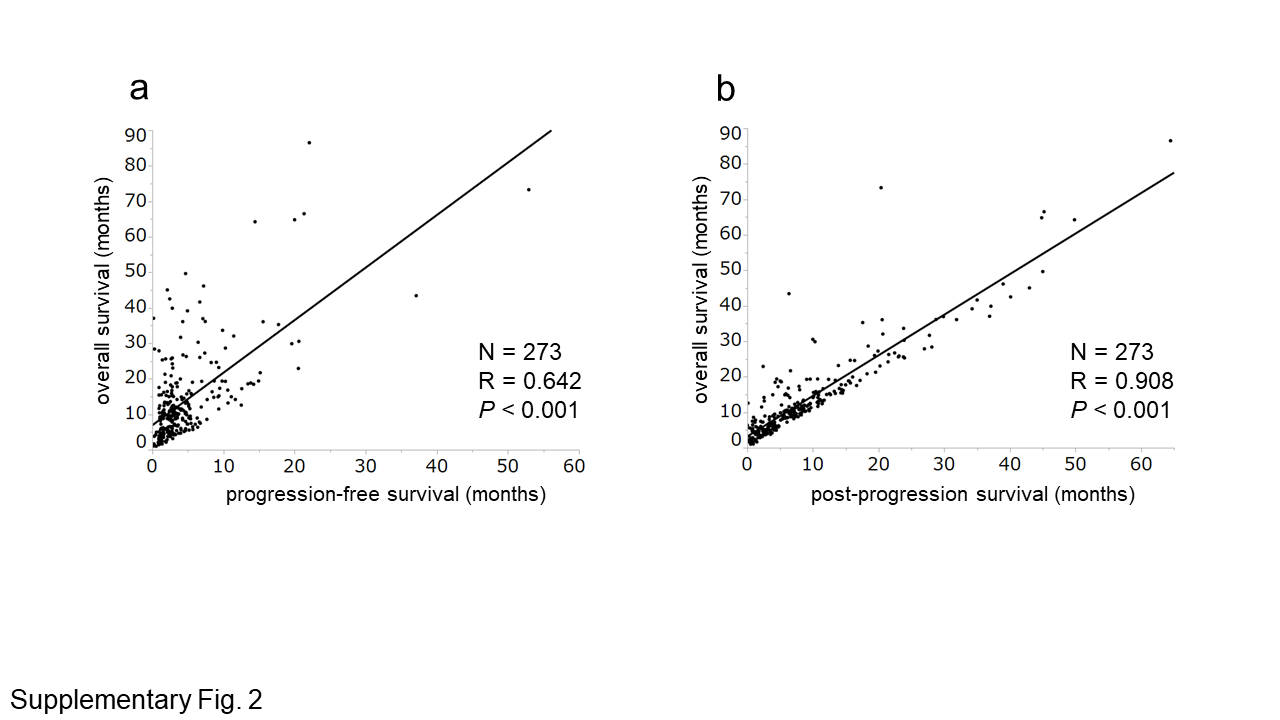

Supplement: Supplementary file 1 [file cancers-13-02247-s001.zip › Fig S2.TIF]

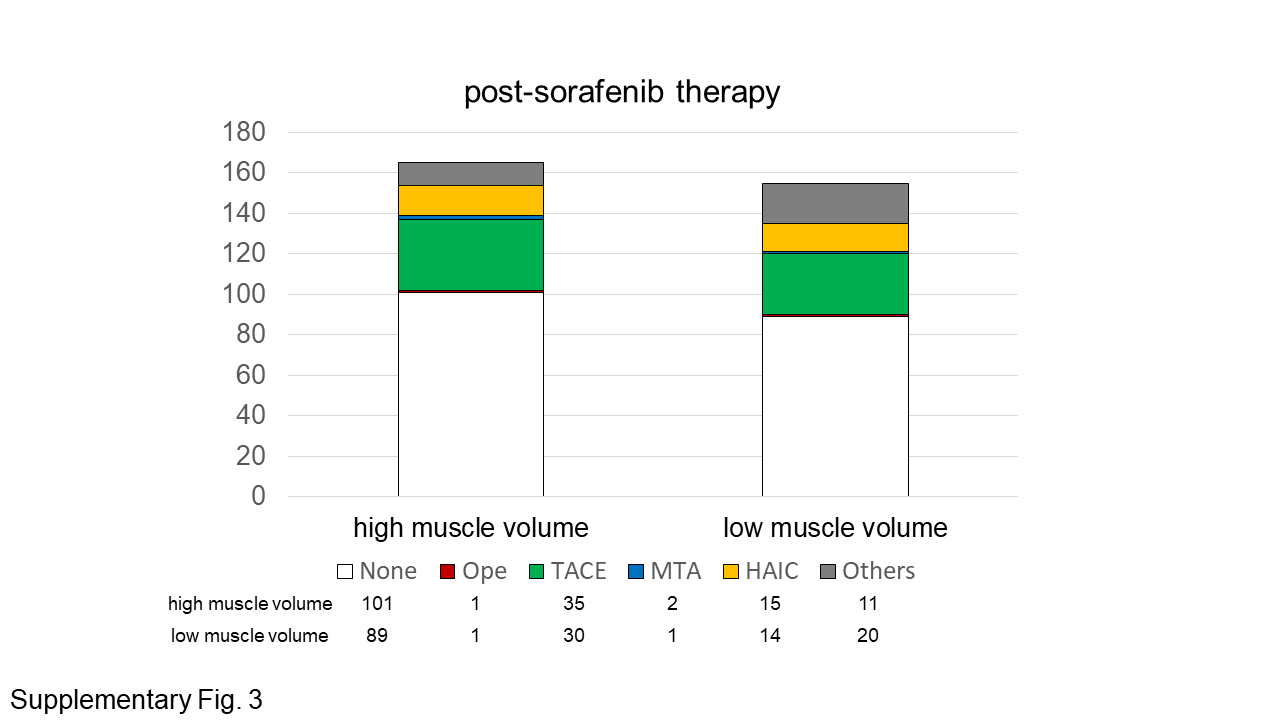

Supplement: Supplementary file 1 [file cancers-13-02247-s001.zip › Fig S3.TIF]

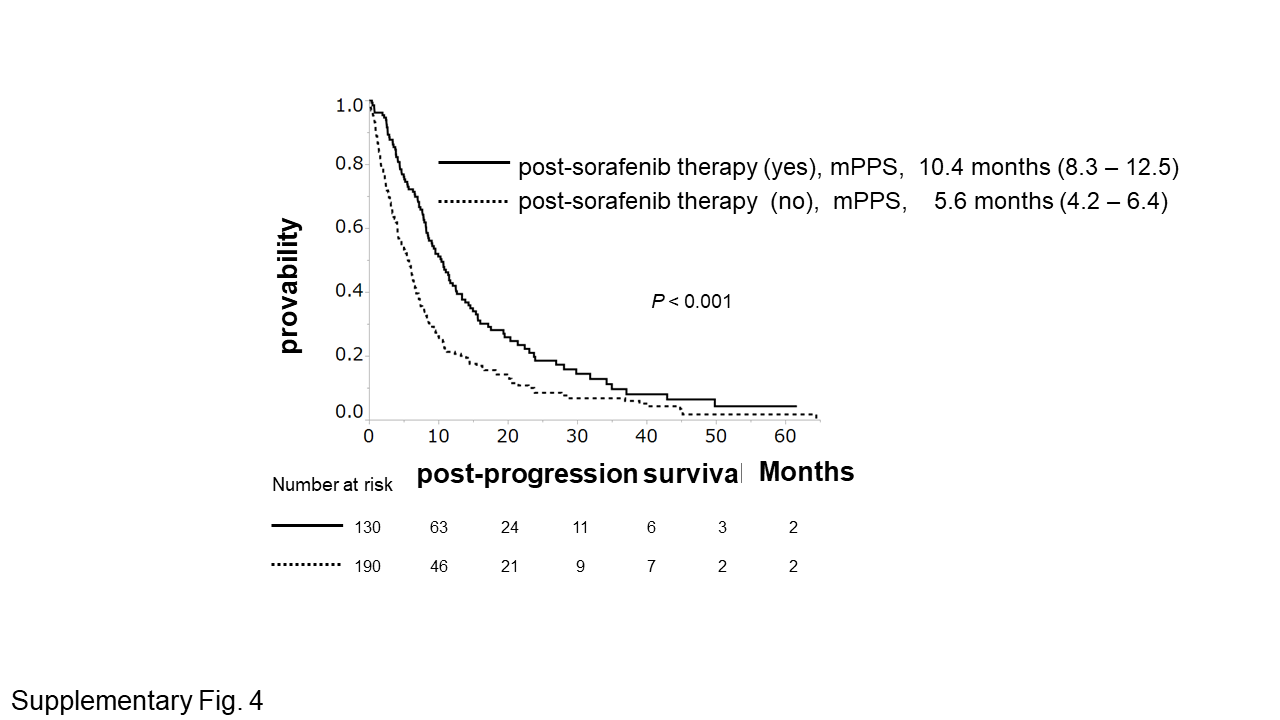

Supplement: Supplementary file 1 [file cancers-13-02247-s001.zip › Fig S4.TIF]

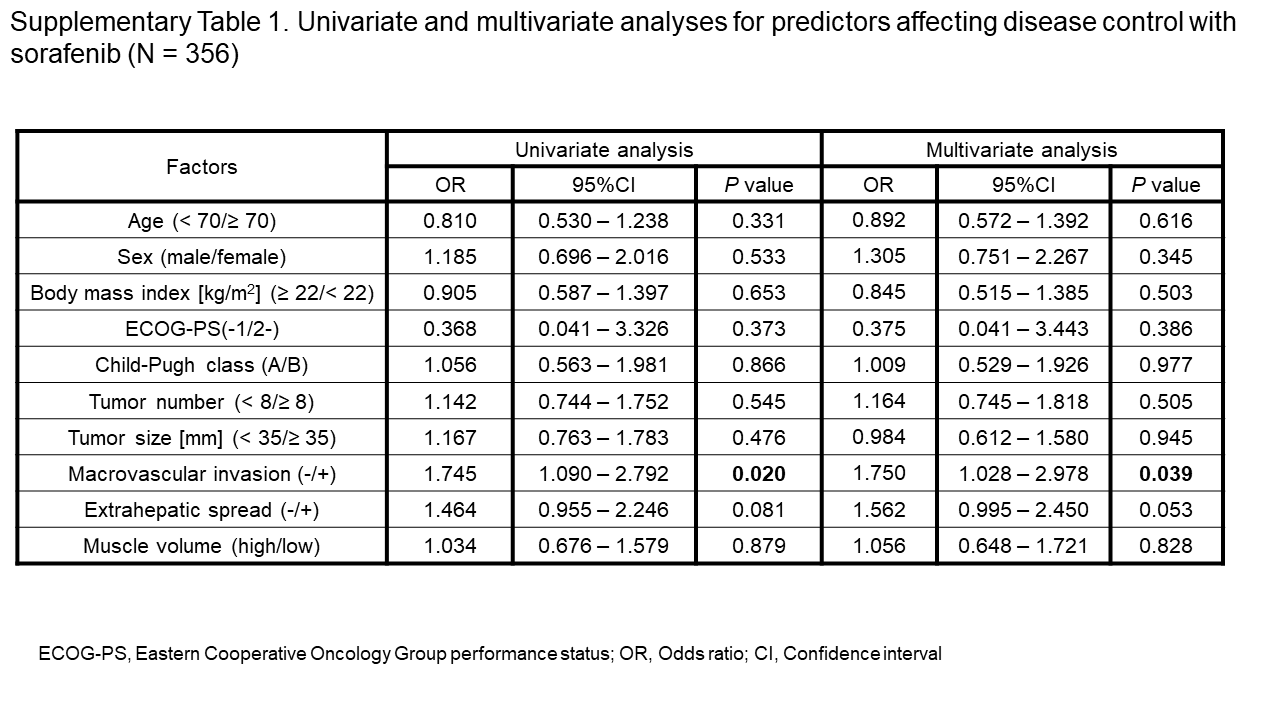

Supplement: Supplementary file 1 [file cancers-13-02247-s001.zip › Table S1.TIF]

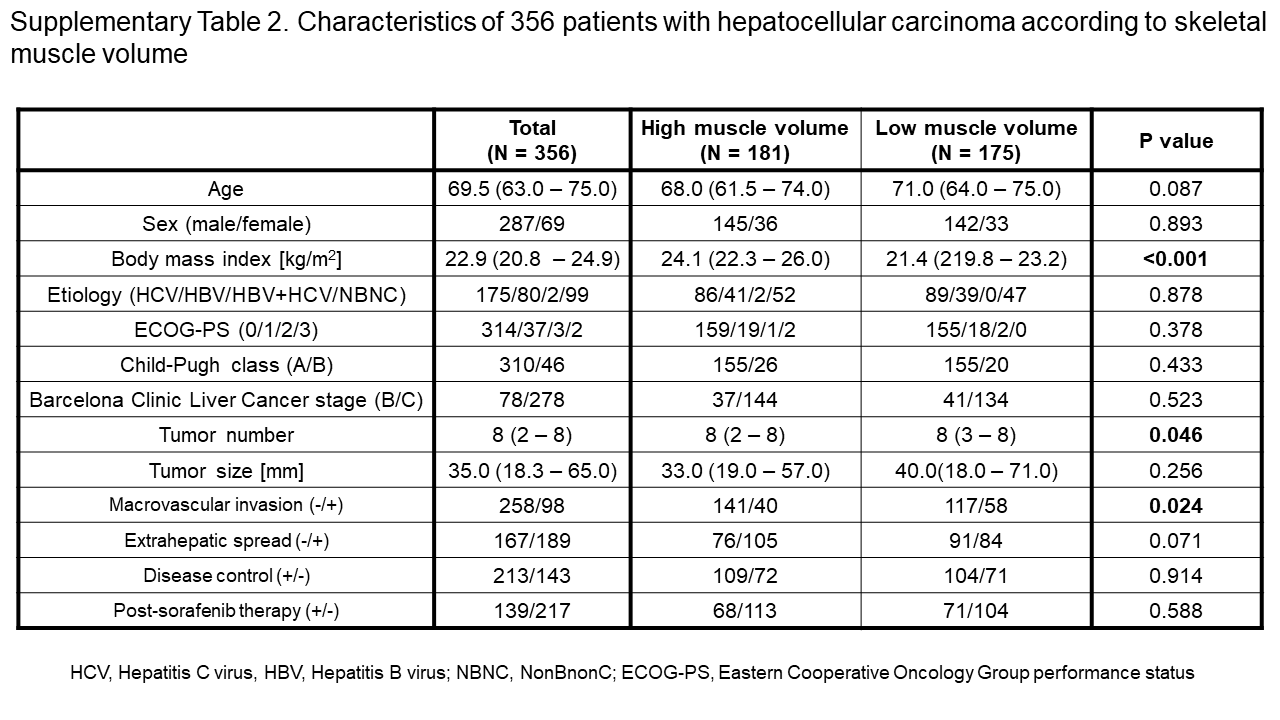

Supplement: Supplementary file 1 [file cancers-13-02247-s001.zip › Table S2.TIF]

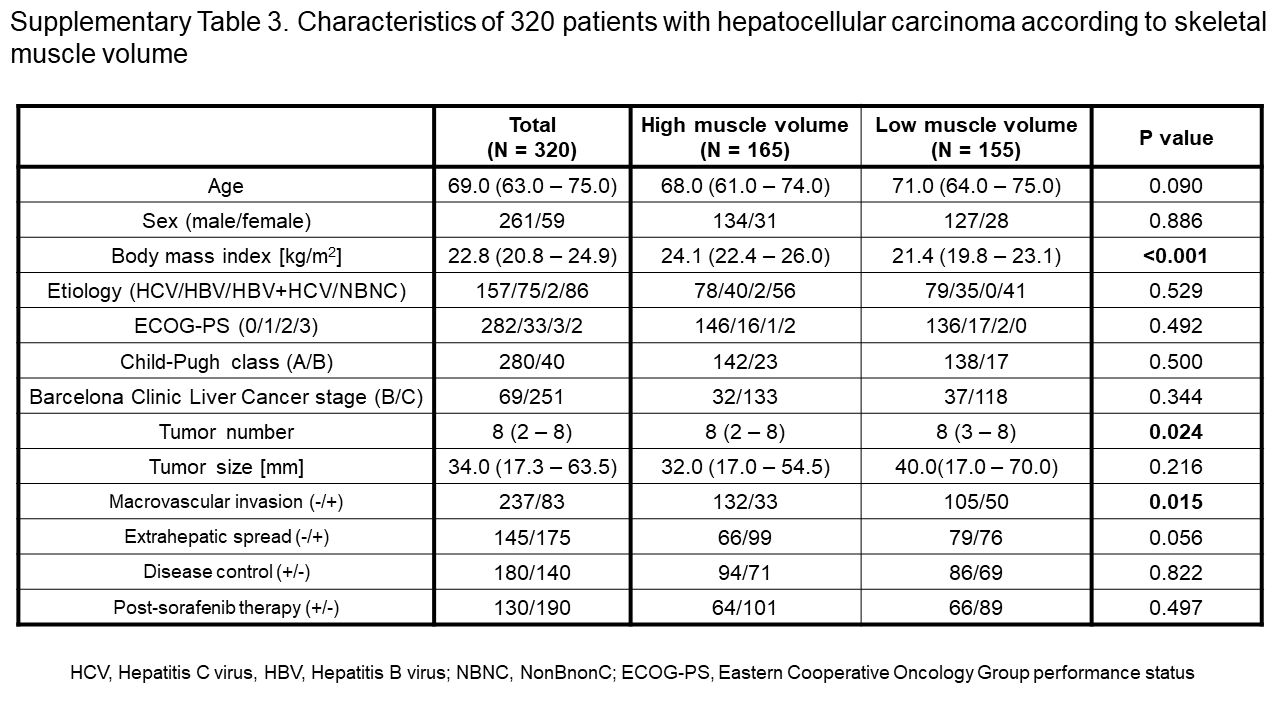

Supplement: Supplementary file 1 [file cancers-13-02247-s001.zip › Table S3.TIF]

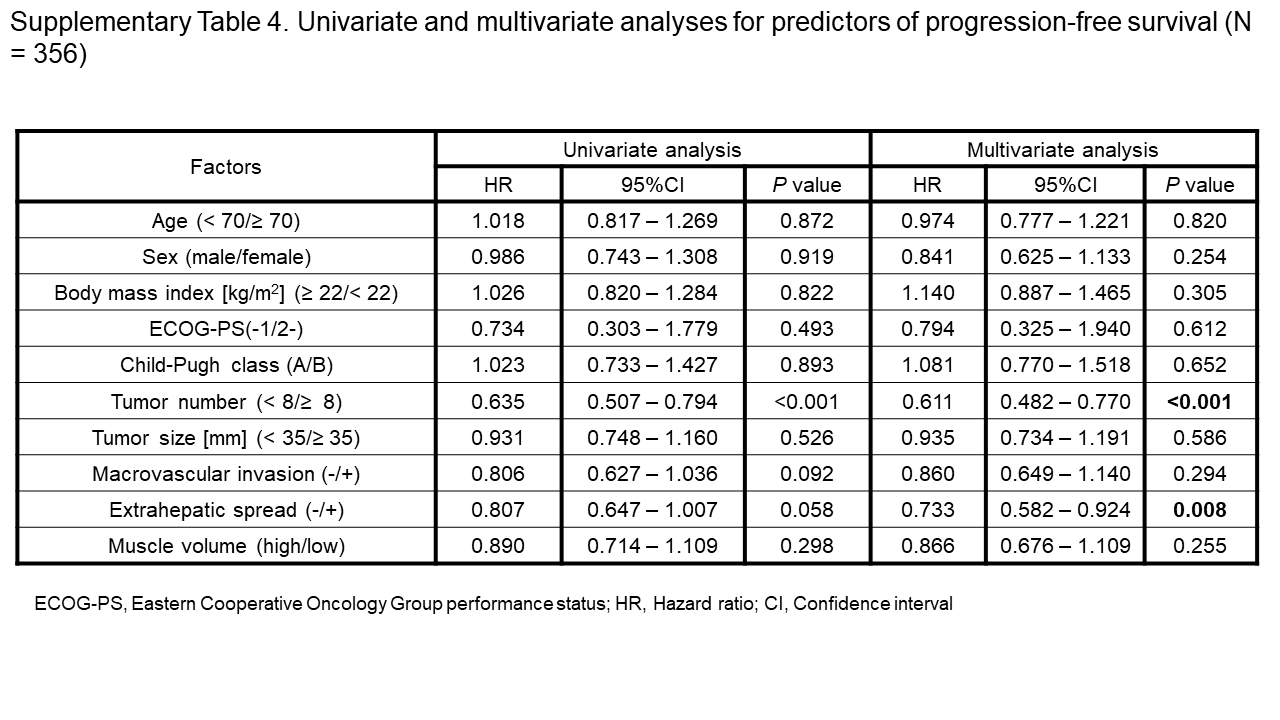

Supplement: Supplementary file 1 [file cancers-13-02247-s001.zip › Table S4.TIF]

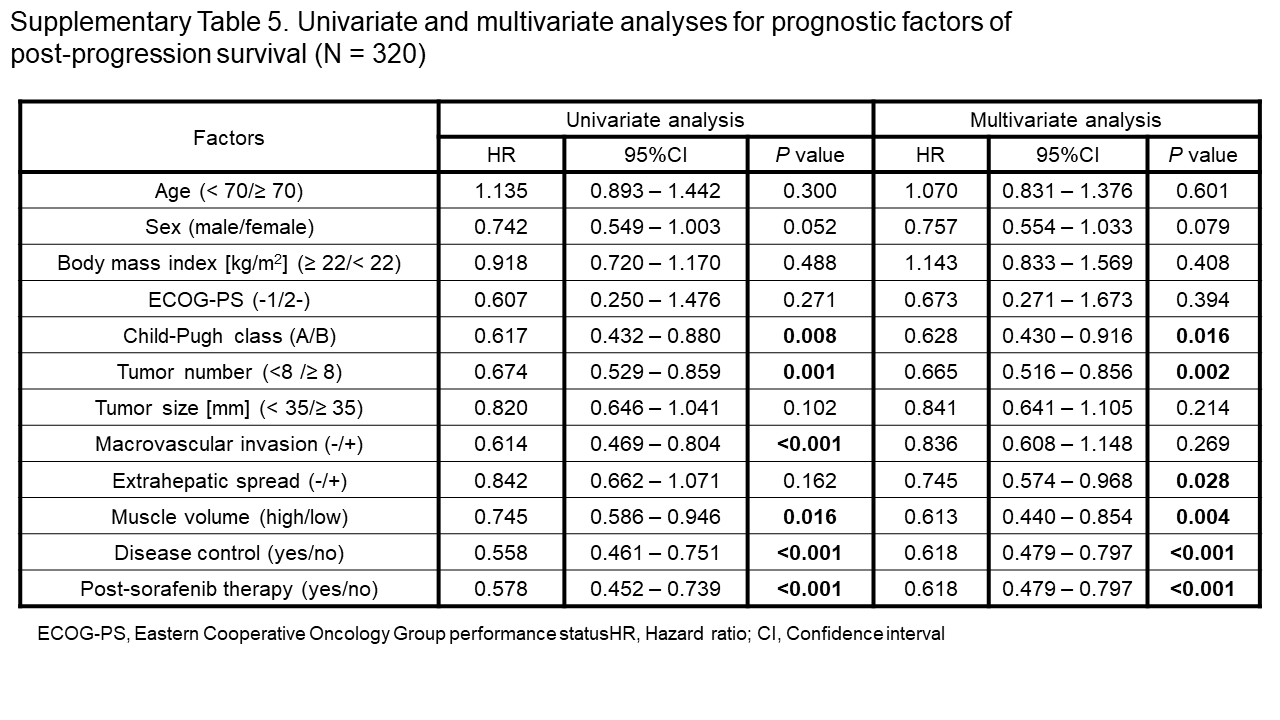

Supplement: Supplementary file 1 [file cancers-13-02247-s001.zip › Table S5.TIF]
